# Necrotizing Enterocolitis in Very Low Birth Weight Infants: A Systemic Review

**DOI:** 10.5402/2012/562594

**Published:** 2012-09-10

**Authors:** Bhoomika K. Patel, Jigna S. Shah

**Affiliations:** Department of Clinical Pharmacy, Shri Sarvajanik Pharmacy College, Near Arvind Baug, Gujarat, Mehsana 384001, India

## Abstract

Necrotizing enterocolitis (NEC) is the most common serious gastrointestinal disorder affecting very preterm or very low birth weight infants. The risk is inversely proportional to gestational age and weight at birth. Fetal growth restriction and compromise may be additional specific risk factors. Despite extensive research and animal studies etiopathogenesis, preventive strategies and management options remain controversial. The present paper reviews the literature for recent advances and newer insights for changing epidemiological trends, pathogenesis, role of inflammatory cytokines, and various preventive and management strategies.

## 1. Introduction

Necrotizing enterocolitis (NEC) is a devastating condition of the neonatal period characterized by bowel necrosis and multisystem organ failure. It is well known that NEC is associated with prematurity and particularly with extremely low birth weight [1–3]. Necrotizing enterocolitis is rare in term infants [4], in whom it is usually associated with congenital anomalies, sepsis, or hypotension [5]. The morbidity and mortality are high, and optimal strategies for treatment remain elusive, despite decades of research.

## 2. Epidemiology

Necrotizing enterocolitis (NEC) is affecting about 5% of all very preterm or very low birth weight infants (VLBW: <1500 g) and about 10% of all extremely preterm or extremely low birth weight (ELBW: <1000 g) infants (Table 1). The rate of NEC-associated acute mortality is generally reported to be greater than 10% overall and more than 25% for infants with NEC severe enough to require a surgical intervention. Infants with NEC have a higher incidence of nosocomial infections and lower levels of nutrient intake, grow more slowly, and have longer durations of intensive care and hospital stay.

Population studies from India on this condition are not widely publicized. In one such report the incidence of NEC in babies less than 32 weeks gestation was 5.2% [6]. Mortality rates vary across centres and range from 10 to 40% depending on gestational age of the baby [7–10]. 

## 3. Risk Factors

1. Prematurity (<28 weeks).
2. Enteral feeding (90% are fed enterally).
3. Growth restricted neonate [11].
4. Maternal hypertensive disease of pregnancy.
5. Placental abruption.
6. Absent or reversed end diastolic flow velocity.
7. Use of umbilical catheters [12–14].
8. Low Apgar scores [12–14].
9. Packed cell transfusions.

## 4. Factors Making Premature Infant's Gut Susceptible to NEC

1. Mechanical factors (barrier integrity):
  - decreased peristalsis,
  - mucus layer deficiency,
  - composition of lipids (premature gut is more permeable).
2. Bacterial factors:
  - delayed or altered bacterial colonization,
  - paucity of anaerobic bacteria.
3. Miscellaneous:
  - decreased gastric acid production,
  - decreased lactase levels,
  - decreased bile acids (insufficient to form bile micelles).

## 5. Genetic Contribution in NEC

Studies comparing the concordance of disease in monozygotic versus dizygotic twins suggest that familial factors may contribute to the risk of NEC. Association studies have so far failed to detect any specific and substantial genetic risk factors. NEC is a sporadic disease which occurs infrequently in individual centres; this sort of investigation would require a coordinated multinational effort to achieve recruitment of sufficient participants to provide a meaningful analysis.

## 6. Pathogenesis

### 6.1. Disordered Enterocyte Signaling

**Figure 1:**
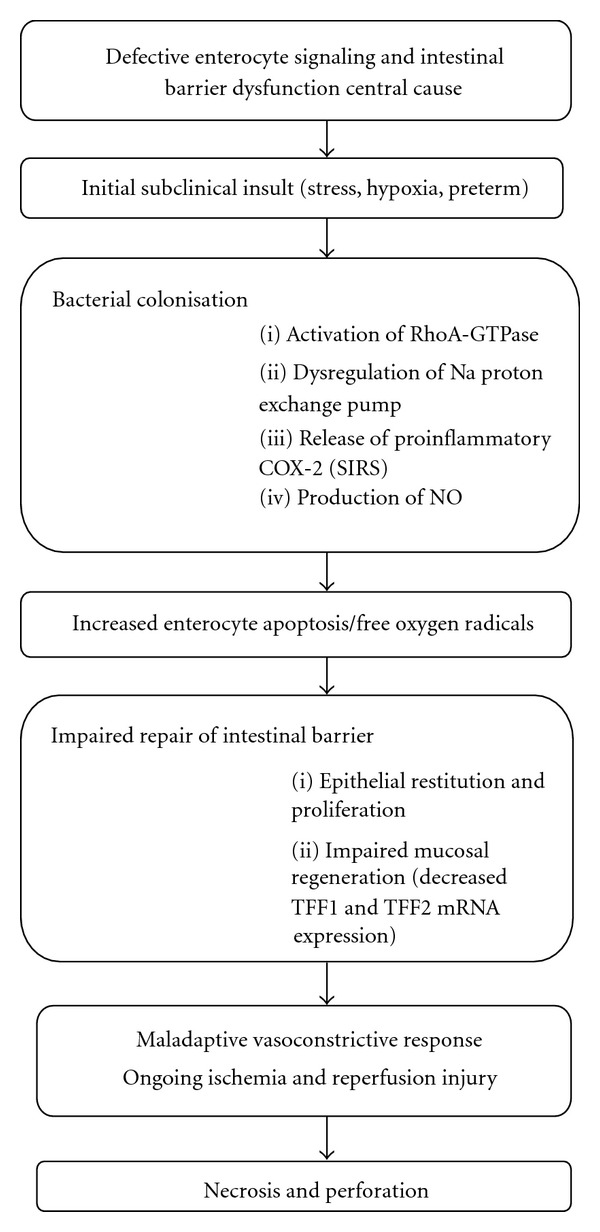
Pathogenesis of NEC.

Recently Hackam et al. have proposed a model of how this can lead to intestinal barrier dysfunction [15]. Rather than serving as an absorptive surface for nutrients, the enterocytes form a tight epithelial barrier that restricts the passage of microbial pathogens and regulates mucosal antigen sampling. Perturbations in the enterocyte signaling can lead to disruption of the epithelial barrier, bacterial translocation, and activation of the inflammatory cascade resulting in full blown NEC [15] (Figure 1). 

### 6.2. Pathophysiologic Mediators

#### 6.2.1. Ischemic Reperfusion Injury

Ischemia causes accumulation of free oxygen radicals generated by the conversion of xanthine dehydrogenase to xanthine oxidase [16]. During reperfusion process there is a further burst of superoxide which causes tissue damage. 

#### 6.2.2. Inflammatory Mediators

Studies show that intestinal cells of premature infants elaborate higher concentrations of proinflammatory cytokines compared to mature cells [17]. IL-18 and IL-12 are upregulated in distal ileum in rat model [18]. IL-10 levels have been shown to be reduced in ileum but increased in serum with babies of NEC [19]. Hepatic inflammatory mediator TNF-*α* suggests a role of gut-bile axis [20]. Epidermal growth factor has maturational effects on intestinal mucosa and its deficiency predisposes infants to NEC [21]. Similarly Platelet activating factor degrading enzyme (PAF-AH) is decreased in neonates with NEC suggesting role of PAF [22]. 

### 6.3. Infective Factors

The epidemics of NEC and isolation of strains of *E. coli* and *Clostridia* as well as improvement in attack rate following the implementation of strict infection control policies and decrease in incidence with prophylactic antibiotics validate the role of infection in the pathogenesis of NEC [23]. In a study on 12 neonates with weekly stool examination by gel electrophoresis 3 neonates who developed NEC have abnormal bands for *Clostridium perfringens* as compared to control infants. In another study on 422 duodenal aspirates collected from 122 VLBW infants no association was found between duodenal colonization with particular strains of *Enterobacteriaceae* and NEC [24]. 

## 7. Preventive Strategies

Various preventive strategies have been tried with an attempt to prevent this disease with high morbidity and mortality. These strategies fall into three categories: those with proven or probable efficacy, those with unproven efficacy or limited data, and experimental strategies.

### 7.1. Breast Milk

The presence of many protective factors in breast milk supports one of the manifold advantages of human milk [25]. Lucas and Cole in a prospective study on 926 preterm infants noted that confirmed NEC was 6- to 10-times more likely in exclusively formula-fed babies than in those who received exclusive human milk and three times more common in those who received formula plus human milk [26]. Meta-analysis of 4 small clinical trials concluded that infants who received donor human milk were 3 times less likely to develop NEC and 4 times less likely to have confirmed NEC [27]. 

### 7.2. Feeding Strategies

#### 7.2.1. Cautious Advancement of Feeds

Cochrane group reviewed 3 good randomized controlled trials comparing slow versus rapid advancement of feeds in preterm neonates receiving parenteral nutrition. There was no significant effect on necrotising enterocolitis. All the three trials were heterogeneous in terms of inclusion criteria (weight) and different definitions used for slow and rapid rates of feeding advancement [28]. 

#### 7.2.2. Trophic Feeding (Minimal Enteral Nutrition)

Cochrane review included 8 studies which were of poor quality in terms of study design, inability to blind the caregivers, and heterogeneity regarding outcome measures and concluded that there was no significant effect on necrotizing enterocolitis.

#### 7.2.3. Standardized Feeding Regimens (SFR)

The Vermont Oxford network “Got Milk” focus group developed eight potentially better practices implementation of which in three NICUs in USA showed reduction in the incidence of NEC [29]. A recent Meta-analysis has reported that introduction of an SFR reduced incidence of NEC by 87% in LBW infants, 43% in VLBW infants, and overall decrease in the incidence by 29%. However these findings need to be interpreted with caution due to heterogeneity across trials and randomized controlled trials are needed to study the efficacy of SFR [30]. 

Interventions for lactation support for mothers of VLBW infants are as follows:

- “Kangaroo” skin-to-skin contact between mother and infant.
- Simultaneous expression of milk from both breasts (using electric pump).
- Peer support in hospital and community.
- Multidisciplinary staff training and continuous professional development to maintain skilled professional support.
- UNICEF “Baby Friendly” accreditation of the associated maternity hospital.

### 7.3. Probiotics

Probiotics are defined as “live microorganisms which when administered in adequate amounts confer a health benefit on the host”. Compared with healthy, full-term infants, the intestinal microbiota in preterm infants features a low number of species, with typically only 3 bacterial species found at 10 days of age [31–33]. Three groups, including enterobacteria such as *E. coli* and *Klebsiella* spp., enterococci such as E. faecalis, and staphylococci such as S. epidermidis, S. aureus, and S. haemolyticus, are the most frequently retrieved [32]. All of these facultative anaerobes persist at high levels in the fecal flora of preterm infants and there is significantly delayed colonization with anaerobes, especially *Bifidobacteria*, compared with that seen in healthy, full-term infants [31–33]. It has been suggested that the enteral administration of probiotics to preterm newborns could prevent infections, prevent NEC, and reduce the use of antibiotics [34]. Some other clinical trials of probiotic preparations to decrease the incidence of neonatal NEC are listed in Table 2.

### 7.4. Prebiotics

The prebiotics are “nondigestible food components that beneficially affect the host by selectively stimulating the growth and/or activity of one or a limited number of bacteria in the colon and thereby improving host health” [35]. Oligosaccharides that are contained in human breast milk are considered to be the prototype of prebiotics, since they have been shown to facilitate the growth of bifidobacteria and lactobacilli in the colon of breast-fed neonates [36–38]. Based on evidence obtained in a search up to January 2004, the committee concluded that only limited studies have evaluated the effects of the addition of prebiotic substances to dietetic products for infants. The committee stated that although the administration of prebiotic oligosaccharides has the potential to increase the total number of bifidobacteria in the feces of infants (including preterm infants), the effects of such administration on different bifidobacteria strains or on different pathogenic bacteria has not been reported. By searching the Cochrane Central Register of Controlled Trials (CENTRAL), MEDLINE, EMBASE, and CINAHL databases and proceedings of relevant conferences, the authors identified 4 RCTs that qualified for inclusion in the paper [39–42]. A total of only 126 preterm infants were included in the paper. The prebiotic oligosaccharides used in these studies were fructooligosaccharides in one RCT [42] and galactooligosaccharides/fructooligosaccharides in 3 RCTs [39–41]. The duration of supplementation ranged from 14 to 30–33 days. Authors of 2 RCTs [40, 41] reported that NEC did not occur in any of their infants. Authors of the other 2 RCTs did not report data related to NEC or sepsis. Meta-analysis of the data from the 2 trials that evaluated stool flora showed a statistically significant increase in bifidobacterial counts in the prebiotic-supplemented group compared with the control group. The authors of the paper concluded that prebiotic-supplemented formula increased stool colony counts of bifidobacteria and lactobacilli in preterm neonates without adversely affecting weight gain. In summary, the quantity and quality of the evidence regarding the effectiveness of the use of specific prebiotics in preterm infants are limited and do not allow one to formulate conclusions regarding the use of prebiotics in clinical practice.

### 7.5. Synbiotics

The term “synbiotic” is used “when a product contains both probiotics and prebiotics” [43]. In a recently published RCT [44], 90 preterm infants received a dietary supplement containing 2 lactobacillus species plus fructooligosaccharides, a supplement containing several species of *Lactobacilli* and *Bifidobacteria* plus fructooligosaccharides, or placebo twice daily for 28 days or until discharge if earlier. The study found that preterm infants who received the supplement containing several species of lactobacilli and bifidobacteria plus fructooligosaccharides were more likely to become colonized with bifidobacteria. There were no significant differences in weight gain or the content of short-chain fatty acids in the stool between groups.

### 7.6. Antenatal Steroids

Crowley reviewed the literature to assess the effects on fetal and neonatal morbidity and mortality and showed that treatment with antenatal corticosteroids is associated with a reduction in the incidence of RDS and IVH and a trend towards reduction in the incidence of NEC [45]. Possible explanations for the increase in NEC include the increased survival of more immature infants, increased use of antenatal steroids, and perhaps a tendency to institute and advance feeds more rapidly than is prudent, given the improved pulmonary status of these neonates. 

### 7.7. Fluid Restriction

Excess fluid intake has been implicated in the pathogenesis of NEC [46]. Cochrane review which included 3 studies concluded that restricted water intake significantly reduces the risks of morbidities like NEC [47]. 

### 7.8. Prophylactic Enteral Antibiotics

The administration of prophylactic oral antibiotics has been investigated in NEC prevention. Evidence to date indicates that oral antibiotics can reduce NEC incidence [48, 49]. A statistically significant increase in the incidence of colonization with resistant bacteria was also shown. Thus routine use of prophylactic antibiotics cannot be recommended [50]. 

### 7.9. Lactoferrin Supplementation

Lactoferrin, an antimicrobial glycoprotein present in colostrum and breast milk, is a key component of the mammalian innate response to infection. Lactoferrin has broad microbicidal activity against Gram-positive cocci, Gram-negative bacilli, and *Candida* species. VLBW infants have low lactoferrin levels and this deficiency is exacerbated by delay in establishing enteral feeding. A recently published Italian multicentre trial examined whether enteral supplementation with exogenous (bovine) lactoferrin for up to 6 weeks, either alone or in combination with a probiotic *Lactobacillus*, reduced the risk of NEC and invasive nosocomial infection in VLBW infants. The incidence of NEC was decreased in the lactoferrin plus probiotic group only. It is plausible that a more modest independent effect of lactoferrin on the risk of NEC may still exist and further large trials are proposed to investigate this possibility.

### 7.10. Oral Immunoglobulins

Immunoglobulins are one of many possible factors in human milk responsible for its protective effects on NEC. Neonates have decreased immunoglobulin levels, particularly secretory IgA [51]. Cochrane review included five studies heterogeneous in terms of entry criteria of neonates, use of placebo (none versus albumin), type of immunoglobulin use (combination of IgG/IgA, only IgG, IgG with a trace of IgM and IgA and none using IgA alone), dose of immunoglobulin, and timing of administration [52]. The oral administration of IgG or an IgG/IgA combination did not result in a significant reduction in the incidence of definite NEC. Further trials are needed.

### 7.11. Immunonutrition: Glutamine and Arginine

VLBW infants who develop NEC have lower plasma levels of the amino acids arginine and glutamine compared with gestation comparable infants who do not develop NEC. In animal models of experimental enterocolitis, glutamine supplementation reduces mucosal damage and lowers the risk of invasive infection and death. Glutamine is abundant in human milk but present only in much lower levels in cow milk formula and absent in standard parenteral nutrition solutions. A relative deficiency of arginine leading to inadequate NO production might predispose the premature infant to inadequate tissue NO levels, vasoconstriction, ischemic-reperfusion injury, and ultimately the development of NEC. Amin et al. in a prospective trial on 152 neonates showed that the incidence of NEC was significantly lower in group receiving supplemental arginine with feeds till 28 days compared with group not receiving supplemental arginine with feeds. However, literature is limited to recommend any practice [53]. 

### 7.12. Patent Ductus Arteriosus and Nonsteroidal Anti-inflammatory Agents

Diastolic steal from a patent ductus arteriosus (PDA) leading to splanchnic under perfusion has been implicated as a risk factor for the development of NEC [54]. Cassady et al. in a small randomized trial have shown that early prophylactic ligation of PDA reduces the risk of NEC [55]. Furthermore, meta-analyses of good quality randomized controlled trials of nonsteroidal anti-inflammatory agents for patent ductus arteriosus closures have not detected any significant effects on the incidence of NEC.

### 7.13. Polyunsaturated Fatty Acids Supplements

Long chain fatty acids have been proposed to modulate inflammation and immunity. Recently Carlson has shown reduced incidence of NEC in group supplemented with egg phospholipids [56]. 

### 7.14. Acidification of Gastric Contents

Carrion and Egan have documented that acidifying the feedings of preterm neonates to a pH low enough to inhibit gastric bacterial proliferation significantly lowers the risk of NEC [57]. Evidence exists that the use of histamine-receptor type 2 (H2) blockers to suppress gastric acidity is associated with a higher risk of NEC (and nosocomial infection) in VLBW infants. Given lack of evidence that gastrooesophageal reflux is a cause of apnoea in preterm infants, it is recommended that use of H2 blockers should be restricted until robust evidence that benefits outweigh harmful effects is obtained.

## 8. Surgical Management

Up to 50% of neonates with NEC develop advanced disease that requires operative treatment [58]. Butter et al. have reported an increase in operative rate from 46% in 1990–1994 to 69% in 1995–1999 primarily due to increase in percentage of stage III patients and post-NEC strictures [59]. The indications for surgery include presence of pneumoperitoneum, indicating perforation of the intestine, clinical deterioration despite maximal medical treatment, abdominal mass with intestinal obstruction, and development of intestinal stricture. Relative indications include fixed dilated intestinal loop, presence of portal gas, thrombocytopenia, and rapid fall in platelet count [60]. There are two multicentric prospective trials underway evaluating primary peritoneal drainage and laparotomy for babies with NEC (NET trial in <1000 Gms in UK and NECSTEPS trial in <1500 Gms neonates in USA).

## 9. Conclusion

NEC continues to be one of the most devastating and unpredictable diseases affecting premature infants. It remains a disease of high morbidity and mortality with adverse long-term outcomes. Promising strategies for minimizing NEC that merit further evaluation include the use of prebiotics and probiotics and the use of arginine supplementation. Large multicentre trials within collaborative networks will be needed to address these questions. Hopefully, future studies aimed at understanding premature intestinal defenses, dietary and bacterial influences, and possible genetic predispositions will lead to development of new prevention and treatment strategies. 

## Figures and Tables

**Table 1:** Clinical findings of NEC (Modified Bell Classification).

(I) “Suspected” NEC:	
(i) Temperature instability, apnoea, bradycardia, lethargy	
(ii) Gastric retention, abdominal distention, emesis, blood in stool	
(iii) Normal or intestinal dilation and mild ileus on abdominal radiograph	

(II) “Definite” NEC:	
(i) As above plus	
(ii) Absent bowel sounds ± abdominal tenderness ± abdominal cellulitis or right lower quadrant mass	
(iii) Radiological evidence of intestinal dilation, ileus, or pneumatosis intestinalis ± ascites	

(III) “Advanced” NEC:	
(i) As above plus	
(ii) Hypotension, bradycardia, severe apnoea, respiratory and metabolic acidosis, coagulopathy, or neutropaenia	
(iii) Signs of peritonitis, marked tenderness, and abdominal distention ± radiological evidence	
of intestinal perforation (pneumoperitoneum)	

**Table 2:** Clinical trials of probiotic preparations to decrease the incidence of neonatal NEC.

Study	Site/patient population	Probiotic administration	Decrease in NEC
Hoyos et al. [61]	Bogota Columbia all admitted newborns historical controls singal site-1237 infants	Daily infloran until D/C (*L acidophilus, B. infantis*)	2.9% versus 6.6%

Lin et al. [62]	Taiwan VLBW < 1500 g prospective, masked randomized control trial singal site-367 infants	2X/day infloran until D/C (*L acidophilus, B. infantis*)	1% versus 5.3%

Bin-Nun et al. [63]	Israel VLBW < 1500 g prospective, masked randomized control trial singal site-145 infants	Daily Abc Dophilus until 36 wks postconceptual age (*B. infantis, Strep thermophilus, B. Bifidus*)	1% versus 14%

Lin et al. [64]	Taiwan VLBW < 1500 g prospective, masked randomized control trial multicentre-434 infants	2X/day infloran until D/C (*L acidophilus, B. infantis*)	1.8% versus 6.5%

## References

[2] Blakey JL, Lubitz L, Barnes GL (1982). Development of gut colonisation in pre-term neonates. *Journal of Medical Microbiology*.

[3] Gewolb IH, Schwalbe RS, Taciak VL, Harrison TS, Panigrahi P (1999). Stool microflora in extremely low birthweight infants. *Archives of Disease in Childhood*.

[4] Magne F, Suau A, Pochart P, Desjeux JF (2005). Fecal microbial community in preterm infants. *Journal of Pediatric Gastroenterology and Nutrition*.

[5] Gewolb IH, Schwalbe RS, Taciak VL, Harrison TS, Panigrahi P (1999). Stool microflora in extremely low birthweight infants. *Archives of Disease in Childhood*.

[6] Narang A, Rao R, Bhakoo ON (1993). Neonatal necrotizing enterocolitis an epidemiological study. *Indian Pediatrics*.

[7] Snyder CL, Gittes GK, Patrick Murphy J, Sharp RJ, Ashcraft KW, Amoury RA (1997). Survival after necrotizing enterocolitis in infants weighing less than 1,000 g: 25 years’ experience at a single institution. *Journal of Pediatric Surgery*.

[8] Schullinger JN, Mollitt DL, Vinocur CD (1981). Neonatal necrotizing enterocolitis. Survival, management, and complications: a 25-year study. *American Journal of Diseases of Children*.

[9] Kanto WP, Wilson R, Ricketts RR (1985). Management and outcome of necrotizing enterocolitis. *Clinical Pediatrics*.

[10] Grosfeld JL, Cheu H, Schlatter M, West KW, Rescorla FJ (1991). Changing trends in necrotizing enterocolitis: experience with 302 cases in two decades. *Annals of Surgery*.

[11] Bashiri A, Zmora E, Sheiner E, Hershkovitz R, Shoham-Vardi I, Mazor M (2003). Maternal hypertensive disorders are an independent risk factor for the development of necrotizing enterocolitis in very low birth weight infants. *Fetal Diagnosis and Therapy*.

[12] Sankaran K, Puckett B, Lee DS (2004). Variations in incidence of necrotizing enterocolitis in Canadian neonatal intensive care units. *Journal of Pediatric Gastroenterology and Nutrition*.

[13] Luig M, Lui K (2005). Epidemiology of necrotizing enterocolitis—part II: risks and susceptibility of premature infants during the surfactant era: a regional study. *Journal of Paediatrics and Child Health*.

[14] Guthrie SO, Gordon PV, Thomas V, Thorp JA, Peabody J, Clark RH (2003). Necrotizing enterocolitis among neonates in the United States. *Journal of Perinatology*.

[15] Hackam DJ, Upperman JS, Grishin A, Ford HR (2005). Disordered enterocyte signaling and intestinal barrier dysfunction in the pathogenesis of necrotizing enterocolitis. *Seminars in Pediatric Surgery*.

[16] Papparella A, Deluca FG, Oyer CE, Pinar H, Stonestreet BS (1997). Ischemia-reperfusion injury in the intestines of newborn pigs. *Pediatric Research*.

[17] Nanthakumar NN, Fusunyan RD, Sanderson I, Walker WA (2000). Inflammation in the developing human intestine: a possible pathophysiologic contribution to necrotizing enterocolitis. *Proceedings of the National Academy of Sciences of the United States of America*.

[18] Halpern MD, Holubec H, Dvorakova K (2002). Up-regulation of IL-18 and IL-12 in the ileum of neonatal rats with necrotizing enterocolitis. *Pediatric Research*.

[19] Edelson MB, Bagwell CE, Rozycki HJ (1999). Circulating pro- and counterinflammatory cytokine levels and severity in necrotizing enterocolitis. *Pediatrics*.

[20] Halpern MD, Holubec H, Dominguez JA, Meza YG, Williams CS, Ruth MC (2005). Hepatic inflammatory mediators contribute to intestinal damage in Necrotisingenterocolitis. *Journal of Physiology, Gastrointestinal and Liver Physiology*.

[21] Pollack PF, Goda T, Colony PC (1987). Effects of enterally fed epidermal growth factor on the small and large intestine of the suckling rat. *Regulatory Peptides*.

[22] Caplan MS, Lickerman M, Adler L, Dietsch GN, Yu A (1997). The role of recombinant platelet-activating factor acetylhydrolase in a neonatal rat model of necrotizing enterocolitis. *Pediatric Research*.

[23] Lee JS, Polin RA (2003). Treatment and prevention of necrotizing enterocolitis. *Seminars in Neonatology*.

[24] Hoy CM, Wood CM, Hawkey PM, Puntis JWL (2000). Duodenal microflora in very-low-birth-weight neonates and relation to necrotizing enterocolitis. *Journal of Clinical Microbiology*.

[25] Undergrove K (2004). Necrotisingenterocolitis: the evidence for the use of human milk in prevention and treatment. *Journal of Human Lactation*.

[26] Lucas A, Cole TJ (1990). Breast milk and neonatal necrotising enterocolitis. *Lancet*.

[27] McGuire W, Anthony MY (2003). Donor human milk versus formula for preventing necrotising enterocolitis in preterm infants: systematic review. *Archives of Disease in Childhood*.

[28] Kennedy KA, Tyson JE, Chamnanvanakij S (2000). Rapid versus slow rate of advancement of feedings for promoting growth and preventing necrotizing enterocolitis in parenterally fed low-birth-weight infants. *Cochrane Database of Systematic Reviews*.

[29] Kuzma-O’Reilly B, Duenas ML, Greecher C (2003). Evaluation, development, and implementation of potentially better practices in neonatal intensive care nutrition. *Pediatrics*.

[30] Patole SK, De Klerk N (2005). Impact of standardised feeding regimens on incidence of neonatal necrotising enterocolitis: a systematic review and meta-analysis of observational studies. *Archives of Disease in Childhood*.

[31] Blakey JL, Lubitz L, Barnes GL (1982). Development of gut colonisation in pre-term neonates. *Journal of Medical Microbiology*.

[32] Gewolb IH, Schwalbe RS, Taciak VL, Harrison TS, Panigrahi P (1999). Stool microflora in extremely low birthweight infants. *Archives of Disease in Childhood*.

[33] Magne F, Suau A, Pochart P, Desjeux JF (2005). Fecal microbial community in preterm infants. *Journal of Pediatric Gastroenterology and Nutrition*.

[34] Caplan MS, Jilling T (2000). Neonatal necrotizing enterocolitis: possible role of probiotic supplementation. *Journal of Pediatric Gastroenterology and Nutrition*.

[35] Gibson GR, Roberfroid MB (1995). Dietary modulation of the human colonic microbiota: introducing the concept of prebiotics. *Journal of Nutrition*.

[36] Adiv OE, Berant M, Shamir R (2004). New supplements to infant formulas. *Pediatric Endocrinology Reviews*.

[37] Dai D, Walker WA (1999). Protective nutrients and bacterial colonization in the immature human gut. *Advances in Pediatrics*.

[38] Quigley EMM, Quera R (2006). Small intestinal bacterial overgrowth: roles of antibiotics, prebiotics, and probiotics. *Gastroenterology*.

[39] Boehm G, Lidestri M, Casetta P (2002). Supplementation of a bovine milk formula with an oligosaccharide mixture increases counts of faecal bifidobacteria in preterm infants. *Archives of Disease in Childhood*.

[40] Mihatsch W, Hoegel J, Pohlandt F (2006). Prebiotic oligosaccharides reduce stool viscosity and accelerate gastrointestinal transport in preterm infants. *Acta Paediatrica*.

[41] Indrio F, Riezzo G, Montagna O, Valenzano E, Mautone A, Boehm G (2007). Effect of a prebiotic mixture
of short chain galacto-oligosaccharides
and long chain fructo-oligosaccharides on gastric
motility in preterm infants. *Journal of Pediatric Gastroenterology and Nutrition*.

[42] Kapiki A, Costalos C, Oikonomidou C, Triantafyllidou A, Loukatou E, Pertrohilou V (2007). The effect of a fructo-oligosaccharide supplemented formula on gut flora of preterm infants. *Early Human Development*.

[43] Schrezenmeir J, De Vrese M (2001). Probiotics, prebiotics, and synbiotics—approaching a definition. *American Journal of Clinical Nutrition*.

[44] Underwood MA, Salzman NH, Bennett SH (2009). A randomized placebo-controlled comparison of 2 prebiotic/probiotic combinations in preterm infants: impact on weight gain, intestinal microbiota, and fecal short-chain fatty acids. *Journal of Pediatric Gastroenterology and Nutrition*.

[45] Crowley P, Chalmers I, Keirse MJNC (1990). The effects of corticosteroid administration before preterm delivery: an overview of the evidence from controlled trials. *British Journal of Obstetrics and Gynaecology*.

[46] Bell EF, Warburton D, Stonestreet BS, Oh W (1979). High-volume fluid intake predisposes premature infants to necrotising enterocolitis. *Lancet*.

[47] Bell EF, Acarregui MJ (2000). Restricted versus liberal water intake for preventing morbidity and mortality in preterm infants. *Cochrane Database of Systematic Reviews*.

[48] Egan EA, Nelson RM, Mantilla G, Eitzman DV (1977). Additional experience with routine use of oral kanamycin prophylaxis for necrotizing enterocolitis in infants under 1,500 grams. *The Journal of Pediatrics*.

[49] Siu YK, Ng PC, Fung SCK (1998). Double blind, randomised, placebo controlled study of oral vancomycin in prevention of necrotising enterocolitis in preterm, very low birthweight infants. *Archives of Disease in Childhood*.

[50] Bury RG, Tudehope D (2000). Enteral antibiotics for preventing necrotising enterocolitis in low birthweight or preterm infants. *Cochrane Database of Systematic Reviews*.

[51] Burgio GR, Lanzavecchia A, Plebani A (1980). Ontogeny of secretory immunity: levels of secretory IgA and natural antibodies in saliva. *Pediatric Research*.

[52] Foster J, Cole M (2004). Oral immunoglobulin for preventing necrotizing enterocolitis in preterm and low birth-weight neonates. *Cochrane Database of Systematic Reviews*.

[53] Amin HJ, Zamora SA, McMillan DD (2002). Arginine supplementation prevents necrotizing enterocolitis in the premature infant. *Journal of Pediatrics*.

[54] Ryder RW, Shelton JD, Guinan ME (1980). Necrotisingenterocolitis: a prospective multicenter investigation. *American Journal of Epidemiology*.

[55] Cassady G, Crouse DT, Kirklin JW (1989). A randomized, controlled trial of very early prophylactic ligation of the ductus arteriosus in babies who weighed 1000 g or less at birth. *New England Journal of Medicine*.

[56] Carlson SE, Montalto MB, Ponder DL, Werkman SH, Korones SB (1998). Lower incidence of necrotizing enterocolitis in infants fed a preterm formula with egg phospholipids. *Pediatric Research*.

[57] Carrion V, Egan EA (1990). Prevention of necrotisingenterocolitis. *Journal of Pediatric Gastroenterology and Nutrition*.

[58] Kosloske AM (1985). Surgey of necrotisingenterocolitis. *World Journal of Surgery*.

[59] Bütter A, Flageole H, Laberge JM (2002). The changing face of surgical indications for necrotizing enterocolitis. *Journal of Pediatric Surgery*.

[60] Pierro A (2005). The surgical management of necrotising enterocolitis. *Early Human Development*.

[61] Hoyos AB (1999). . Reduced incidence of necrotizing enterocolitis associated with enteral administration of Lactobacillus acidophilus and Bifidobacterium infantis to neonates in an intensive care unit.. *International Journal of Infectious Diseases*.

[62] Lin HC, Su BH, Chen AC, Lin TW, Tsai CH, Yeh TF (2005). Oral probiotics reduce the incidence and severity of necrotizing enterocolitis in very low birth weight infants. *Pediatrics*.

[63] Bin-Nun A, Bromiker R, Wilschanski M, Kaplan M, Rudensky B, Caplan M (2005). Oral probiotics prevent necrotizing enterocolitis in very low birth weight neonates. *Journal of Pediatrics*.

[64] Lin HC, Hsu CH, Chen HL, Chung MY, Hsu JF, Lien RI (2008). Oral probiotics prevent necrotizing enterocolitis in very low birth weight preterm
infants: a multicenter, randomized, controlled trial. *Pediatrics*.

